# Peptide Inhibitor of Complement C1, RLS-0071, Reduces Zosteriform Spread of Herpes Simplex Virus Type 1 Skin Infection and Promotes Survival in Infected Mice

**DOI:** 10.3390/v13081422

**Published:** 2021-07-22

**Authors:** Maimoona S. Bhutta, Daniel G. Sausen, Kirstin M. Reed, Elisa S. Gallo, Pamela S. Hair, Brittany P. Lassiter, Neel K. Krishna, Kenji M. Cunnion, Ronen Borenstein

**Affiliations:** 1 Department of Microbiology and Molecular Cell Biology, Eastern Virginia Medical School, Norfolk, VA 23507, USA; BhuttaM@EVMS.EDU (M.S.B.); SausenDG@EVMS.EDU (D.G.S.); ReedKM@EVMS.EDU (K.M.R.); cunniokm@EVMS.EDU (K.M.C.); 2 Board-Certified Dermatologist and Independent Researcher, Norfolk, VA 23507, USA; 3 ReAlta Life Sciences, Inc., Norfolk, VA 23502, USA; phair@realtals.com (P.S.H.); blassiter@realtals.com (B.P.L.); nkrishna@realtals.com (N.K.K.); 4 Department of Pediatrics, Eastern Virginia Medical School, Norfolk, VA 23507, USA; 5 Children’s Specialty Group, 811 Redgate Avenue, Norfolk, VA 23507, USA; 6 Children’s Hospital of The King’s Daughters, Norfolk, VA 23507, USA

**Keywords:** RLS-0071, PIC1, antiviral, herpes simplex type 1, acyclovir-resistance, zosteriform infection, anti-inflammatory, complement, neutrophil, wound healing

## Abstract

Herpes simplex virus type 1 (HSV-1) is a prevalent human pathogen primarily transmitted through skin-to-skin contact, especially on and around mucosal surfaces where there is contact with contaminated saliva during periods of viral shedding. It is estimated that 90% of adults worldwide have HSV-1 antibodies. Cutaneous HSV-1 infections are characterized by a sensation of tingling or numbness at the initial infection site followed by an eruption of vesicles and then painful ulcers with crusting. These symptoms can take ten days to several weeks to heal, leading to significant morbidity. Histologically, infections cause ballooning degeneration of keratinocytes and formation of multinucleated giant cells, ultimately resulting in a localized immune response. Commonly prescribed treatments against HSV-1 infections are nucleoside analogs, such as acyclovir (ACV). However, the emergence of ACV-resistant HSV (ACV^R^-HSV) clinical isolates has created an urgent need for the development of compounds to control symptoms of cutaneous infections. RLS-0071, also known as peptide inhibitor of complement C1 (PIC1), is a 15-amino-acid anti-inflammatory peptide that inhibits classical complement pathway activation and modulates neutrophil activation. It has been previously shown to aid in the healing of chronic diabetic wounds by inhibiting the excessive activation of complement component C1 and infiltration of leukocytes. Here, we report that treatment of cutaneous infections of HSV-1 and ACV^R^-HSV-1 in BALB/cJ mice with RLS-0071 significantly reduced the rate of mortality, decreased zosteriform spread, and enhanced the healing of the infection-associated lesions compared to control-treated animals. Therefore, RLS-0071 may work synergistically with other antiviral drugs to aid in wound healing of HSV-1 cutaneous infection and may potentially aid in rapid wound healing of other pathology not limited to HSV-1.

## 1. Introduction

An estimated 3.7 billion individuals live with herpes simplex virus 1 (HSV-1) infection worldwide [1]. HSV-1 primarily infects the mucosal epithelial cells, causing the formation of painful, vesicular lesions [2]. Primary infection in the epithelium occurs as HSV-1 targets the basal keratinocytes, spreading into the supra-basal layers [3,4]. Cellular entry of HSV-1 requires the interaction of envelope glycoproteins and cell surface receptors. Glycoprotein D (gD) receptors, herpesvirus entry mediator (HVEM), nectin-1/nectin-2, and 3-O-sulfotransferase-generated heparan sulfate mediate viral entry in murine and human models of infection [3,5].

Murine models have been widely used to investigate cutaneous HSV-1 infection, as they contain HVEM and nectin-1 receptor homologs that support viral entry [3]. In addition, cutaneous or zosteriform scarification models are utilized to infect mice efficiently. In the latter, HSV-1 is scratched onto the skin to expose the epidermal cells to the virus, and as the virus spreads to the innervating sensory neurons, it travels to the dorsal root ganglia. A retrograde spread of infection is seen following viral reactivation, as HSV-1 travels from the spinal cord back to the skin, causing the formation of zosteriform lesions along the dermatome of the nerve [6,7]. Commonly prescribed antiviral agents against HSV are nucleoside analogues, including acyclovir (ACV), which acts as a competitive inhibitor of viral DNA polymerase to block viral replication [8]. ACV-resistant (ACV^R^)-HSV strains have been isolated at an increasing rate from immunocompromised patients and stem-cell transplant recipients [9]. Due to the growing population of HSV-1 infected individuals, the emergence of resistant viral strains has generated a need to develop new antiviral agents. 

Complement pathways regulate the clearance of necrotic/apoptotic cells, inflammation, and tissue regeneration in response to injury. The complement system is activated by classical, lectin, or alternative pathways and regulates the activation/migration of immune cells, such as neutrophils. Notably, previous research has reported that keratinocytes contain abundant innate immune mediators, complement receptors, and regulatory proteins [10,11]. However, elevated levels of complement factors (C3, C5, and membrane attack complex (MAC)) have been reported to cause excessive inflammation, thus delaying the process of healing and leading to the formation of chronic wounds [10,11]. Therefore, inhibition of complement activation may improve the healing process. 

Peptide inhibitor of complement C1 (RLS-0071) is a 15-amino-acid peptide with a monodisperse 24-mer polyethylene glycol (PEG) on its C terminus (IALILEPICCQERAA-dPEG24) that inhibits the activation of C1 and the classical complement pathway and modulates neutrophil activation via inhibition of myeloperoxidase activity and neutrophil extracellular trap formation [12,13,14,15]. RLS-0071 was previously reported to bind to C1q and mannose-binding lectin (MBL), displacing the serine protease complex and preventing C1 cleavage [12]. Recently, direct topical application of RLS-0071 was shown to reduce inflammation associated with diabetic wounds of db/db mice, suggesting that this compound may play a role in reducing complement system activation and infiltration of immune cells in the wounded skin [16].

In this paper, we report that RLS-0071 has beneficial activity against HSV-1 skin infection in BALB/cJ mice. Although RLS-0071 did not show direct in vitro HSV-1 inhibition, RLS-0071 formulated in 2.5% hydroxyethyl cellulose (HEC) gel resulted in a significant reduction in mortality and infection scores compared to vehicle-control of HSV-1 and ACV^R^-HSV-1 skin infection in BALB/cJ mice. Furthermore, we propose that RLS-0071 inhibits the activation of C1 in surface wounds of BALB/cJ mice, thus reducing inflammation and promoting wound healing. 

## 2. Materials and Methods 

### 2.1. Cells and Animals 

GFP-HSV-1 strain 17+ (a generous gift from Dr. Peter O’Hare [17]), was propagated in Vero cells (CCL-81, ATCC) in Dulbecco’s Modified Eagle Medium (DMEM, Cat# sc-224478, Ultra-Cruz, Dallas, TX, USA) supplemented with 5% heat-inactivated fetal bovine serum (FBS, Cat# 10082-147, Gibco, Waltham, MA, USA) and 1% penicillin and streptomycin (P/S, Cat# 15140-122, Gibco, Waltham, USA), DMEM/5%. Acyclovir-resistant GFP-HSV-1 (ACV^R^-HSV-1) mutant strain was generated in laboratory [18]. Female BALB/cJ mice (5–6 weeks old; Jackson Laboratory, Bar Harbor, ME, USA) were housed in biosafety level 2 (BSL-2) animal facility. Following infections, all mice were single-housed in sterile cages and kept on a 12:12 light-dark cycle. Eastern Virginia Medical School’s Institutional Animal Care and Use Committee approved all in vivo procedures under protocol #18-012. 

### 2.2. Sequencing ACV^R^-HSV-1 17+

Vero cells were infected with 1MOI of ACV^R^-HSV-1 in medium 199 (1X) (Cat# 11150-059, Gibco), supplemented with 1% FBS (Cat# 10082-147, Gibco) and 1% P/S (Cat# 15140-122, Gibco), referred to as 199V, for 1 h at 37 °C. The infected media was removed, fresh DMEM/5% was added to the cells, and the cells were incubated at 37 °C for 20 h. The infected cells were then harvested and collected using low centrifugation (3000 rpm for 5 min). Viral DNA was extracted from the cells using QIAamp DSP DNA Mini Kit (Cat# 61304, Qiagen, MD, USA) according to the manufacturer’s instructions. The desired thymidine kinase (TK) primers (Table 1) were generated using ApE Software (ApE- A plasmid Editor, version 3.0.3, Multiplatform DNA editing software, Salt Lake City, UT, USA) and human herpesvirus 1 strain 17, complete genome (NCBI Reference Sequence: NC_001806.2). 

The forward primer was located at nucleotides 47,886-47,868 of HSV-1 strain 17+ genome (NCBI Reference Sequence: NC_001806.2), 83 nucleotides upstream to the ORF of the TK gene. The reverse primer was located at nucleotides 46,598-46,617 of HSV-1 strain 17+ genome (NCBI Reference Sequence: NC_001806.2), 75 nucleotides upstream to the ORF of the TK gene. The entire TK gene (1131 bp plus the additional 83 bp and 75 bp) was amplified using Herculase II PCR Fusion Polymerase Kit (Cat# 600675, Agilent Technologies Inc., Santa Clara, CA, USA) according to the manufacturer’s instructions. The cycling program consisted of initial denaturing for 3 min at 95 °C, followed by 34 cycles of 30 s at 95 °C, 30 s at 51 °C, and 1.5 min at 72 °C, with a final extension of 5 min at 72 °C. The PCR product was purified using GeneJet PCR Purification kit (Cat# K0701, Thermo Fisher Scientific, Waltham, MA, USA) according to the manufacturer’s instructions. The purified PCR product was sequenced, using the primers in Table 1, in forward and reverse directions by the Molecular Core Facility at Eastern Virginia Medical School (Norfolk, VA, USA). 

### 2.3. Plaque Assay

The virus titer of GFP-HSV-1 (6.0 × 10^7^ PFU/mL) was determined by standard plaque assays, as previously described by our lab [18,19]. The antiviral and virucidal activity of RLS-0071, following GFP-HSV-1 infection, was assessed in in vitro cell culture before in vivo experiments. The Vero cell monolayer in a 6-well plate was pretreated with 0-to-5 mM of RLS-0071 or 0.05 M Histidine Buffer (HIS buffer) for 1 h. The cells were then infected with 0.1 MOI of GFP-HSV-1 17+ in 199 media, supplemented with 1% FBS and 1% P/S (199V), for 1 h at 37 °C. After incubation, Vero cells were washed twice with DPBS 1× and replaced with 199V media containing the respective treatments for 16 h. To test the virucidal activity of RLS-0071, 0.1 MOI cell-free GFP-HSV-1 17+ virus was incubated with increasing concentrations of RLS-0071 or HIS buffer for 1 h. The treated virus was then used to infect Vero cell monolayer in 6-well plates in 199V media for 1 h at 37 °C. After incubation, the Vero cells were washed twice with DPBS 1×, and fresh 199V was added to the monolayer for 16 h. The infected culture was collected, and the viral titer was measured using plaque assays, as previously described [18,19].

### 2.4. Zosteriform Infection Model and Treatments

Prior to infection, the right flank skin of female BALB/cJ mice was chemically denuded using Nair cream (Nair™, Ewing, NJ, USA) under gas anesthesia. Following 24 h with no signs of chemical irritation, BALB/cJ mice were anesthetized with intraperitoneal injections of ketamine/xylazine (120 mg/kg; 12 mg/kg), and the shaved skin was scratch-inoculated with 6 × 10^4^ PFU of GFP-HSV-1 strain 17+ or 2.0 × 10^5^ PFU of ACV^R^-HSV-1 [18], using the pointed-side of a 27-gauge needle. The epidermal scarification-zosteriform model was used as previously described [6].

RLS-0071 was a generous gift from ReAlta Life Sciences (Norfolk, VA, USA) and was solubilized in 0.05M Histidine buffer (HIS buffer) (pH 6.5). To ascertain there was no vehicle effect, a control study was conducted wherein mice (N = 5/treatment) were initially treated with 40% HIS buffer in 2.5% hydroxyethyl cellulose (HEC) gel (Cat# 09368, Sigma-Aldrich, St. Louis, MO, USA) and compared with animals treated with 10% DMSO (Cat#D2650-5X10mL, Sigma-Aldrich, MO, USA) in 2.5% HEC gel. 

The treatment studies, following infections with GFP-HSV-1 17+ or ACV^R^-HSV-1, were designed to treat the inoculated site with 10% DMSO (acyclovir vehicle control) and 40% HIS buffer formulated in 2.5% HEC gel, 14.4 mM RLS-0071 (40 mg/mL) in 2.5% HEC gel, or 10 mM ACV (2.25 mg/mL; Cat#2513, Tocris, MN, USA; control for the standard of care) with 40% HIS buffer in 2.5% HEC gel. The treatment schedule started 1-h post-infection and continued twice daily (at 12-h intervals) for 14 days. Signs of disease at the inoculation site were scored by the appearance of vesicles and erosions, as previously outlined [18]. Table 2 outlines the infection grading scales utilized in this study. 

### 2.5. Statistical Analyses

Prism Graph Pad version 9.0.0 (GraphPad Software, San Diego, CA, USA) was used to analyze survivability by Kaplan–Meier analysis and Log-rank (Mantel–Cox) test. Changes in infections scores were analyzed using one-way ANOVA (with independent *t*-tests) or mixed-model ANOVAs (with multiple comparisons). 

## 3. Results

### 3.1. RLS-0071 Has No In Vitro Antiviral Activity

RLS-0071 has been previously reported to have antimicrobial activity against Pseudomonas aeruginosa, Staphylococcus aureus, Klebsiella pneumoniae, Neisseria meningitidis, Neisseria gonorrhoeae, Gardnerella vaginalis, and Prevotella bivia bacteria [20]. We began by investigating whether RLS-0071 possesses antiviral or virucidal activity against HSV-1 in vitro. To test the antiviral activity of RLS-0071, 80–85% confluent Vero cells were pretreated with varying concentrations of RLS-0071 or HIS buffer (control) and then infected with 0.1 MOI of GFP-HSV-1 17+. The infected media was replaced with fresh 199V media and incubated for an additional 16 h. The viral titer (PFU/mL) was determined through plaque assay. RLS-0071 did not exhibit a reduction in viral titer when compared to HIS Buffer-treated cells (Table S1). Following this, we examined whether RLS-0071 possesses virucidal activity against HSV-1. 0.1 MOI of GFP-HSV-1 cell-free virus, pre-incubated with varying concentrations of RLS-0071 or HIS buffer for 1 h prior to infecting Vero cells. Vero cells were infected with the cell-free virus for 1 h before the infected media was replaced with fresh 199V media. The infected cells were incubated for an additional 16 h, and the viral titer was determined by plaque assay. RLS-0071 did not exhibit a reduction in viral titer when compared to control-treated Vero cells; thus, virucidal effect could not be concluded in vitro (Table S2).

### 3.2. Histidine Buffer and DMSO Are Neutral for HSV-1 Cutaneous Infection

To demonstrate that the RLS-0071 and the ACV carriers have no effect on the infection, 0.05 M histidine buffer (HIS buffer), in which RLS-0071 is soluble, was compared with DMSO, in which ACV is soluble. The inoculated site of BALB/cJ mice was treated with 10% DMSO (control) in 2.5% HEC gel or 40% HIS Buffer (formulation ratio for RLS-0071) in 2.5% HEC gel. The infection scores of the animals were averaged each day across 14 days and analyzed. Our results indicate that there was no significant difference between both control treatments, as they demonstrated a 0% survivability rate within 14 days and a median survival for 9 days (*p* = 0.6630). No observed significance was recorded, as both DMSO-treated and HIS Buffer-treated animals exhibited a significant increase in the severity of HSV-1 infection, reaching the study endpoint (score 7) by day 9 (Figure S1). We concluded that the subsequent observed effects of the treatments would be unaffected by the addition of their respective solvents. 

### 3.3. RLS-0071 Formulated in HEC Gel Protects BALB/cJ Mice Against GFP-HSV-1 Zosteriform Infection

RLS-0071 has previously demonstrated chronic-wound-healing capabilities in db/db mice [16] via inhibition of complement activation and neutrophil extracellular trap (NET) formation in a dose-dependent manner [14]. Thus, we examined the effect of RLS-0071 in healing of infectious wounds in BALB/cJ mice.

Five- to six-week-old female BALB/cJ mice (15 mice in each group) were used to analyze the efficacy of RLS-0071 against zosteriform infection. Cutaneous GFP-HSV-1 infections were conducted using the epidermal scarification-zosteriform model, as previously described by Goel et al. [6]. For topical treatments, we formulated 14.4 mM RLS-0071 (40 mg/mL) in 2.5% hydroxyethyl cellulose (HEC) gel, which does not have active microbicidal activity and has been adopted as a placebo in many clinical trials of microbicides [21,22]. All animals were inoculated with 6.0 × 10^4^ PFU GFP-HSV-1 17+, as previously established [18].

Following GFP-HSV-1 infection, the inoculation site was treated with 10% DMSO (vehicle control) formulated in 2.5% HEC gel, 14.4 mM RLS-0071 in 2.5% HEC gel, or 10 mM acyclovir (ACV) in 2.5% HEC gel (control for the standard of care). Each treatment was administered 1-h post-infection (p.i.) and continued b.i.d. at 12-h intervals for 14 days. Animals were monitored daily for any signs of physical deterioration. Disease at the inoculation site was scored by the appearance of vesicles and erosions.

**Figure 1:**
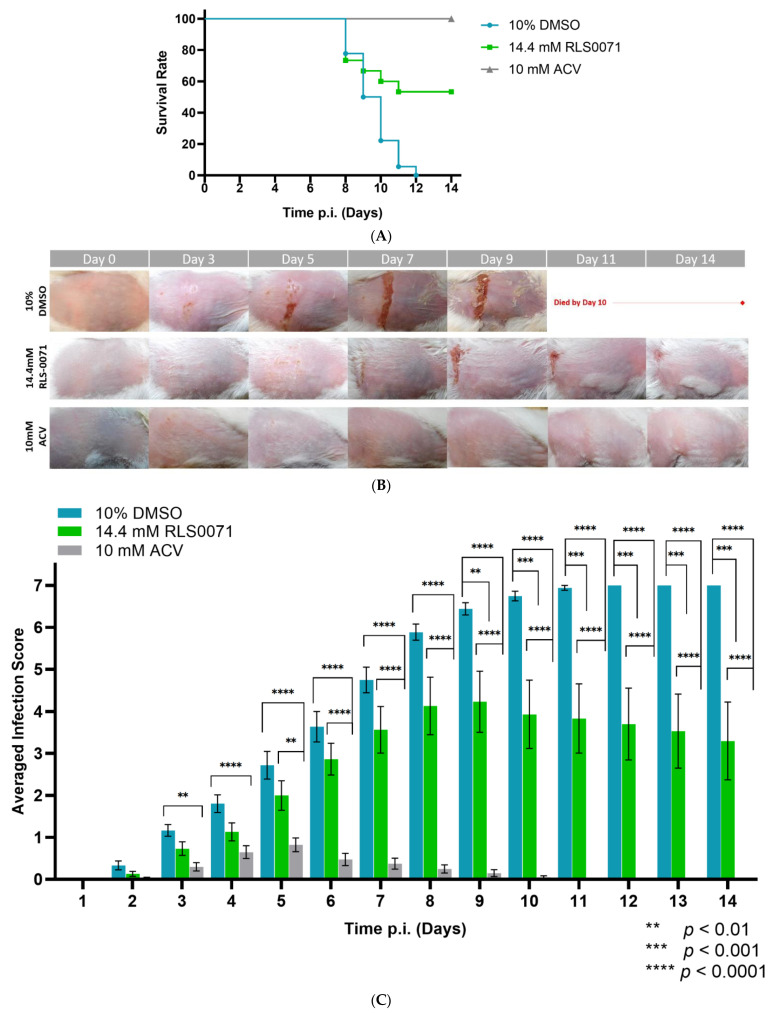

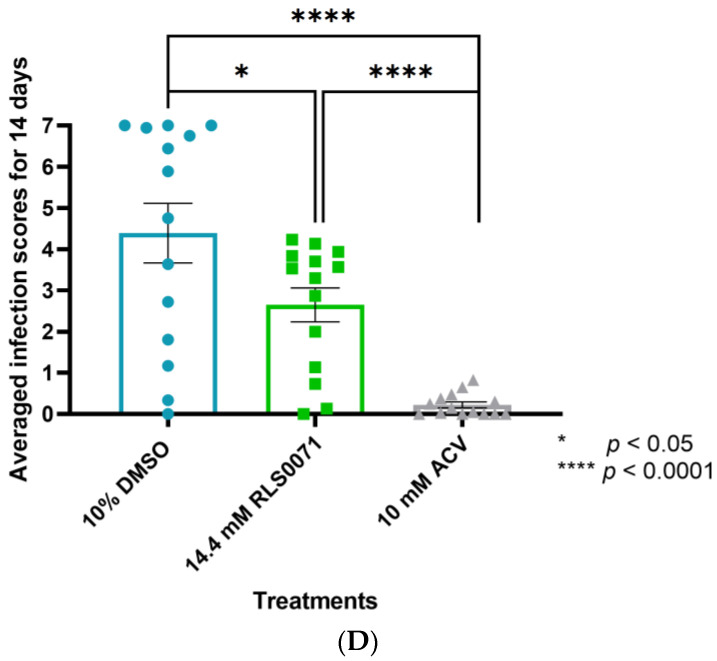
GFP-HSV-1 zosteriform infections in BALB/cJ mice, following the application of 10% DMSO, 14.4 mM RLS-0071, or 10 mM ACV in 2.5% hydroxyethyl cellulose (HEC) gel. (**A**) Age- and weight-matched BALB/cJ mice were inoculated with 6 × 10^4^ PFU of GFP-HSV-1 and received their respective treatments. Animals were monitored for survival for 14 days. (**B**) Representative images of female BALB/cJ mice in an epidermal scarification-zosteriform model receiving respective treatments across varying time points of the study. (**C**) Averaged infection scores of animals were analyzed for each treatment group across 14 days. (**D**) Distribution of averaged infection score of all animals per day for 14 days. RLS-0071-treated animals demonstrated a significant reduction in the appearance of vesicles and erosions on the skin of BALB/cJ mice compared to control animals. (**C**) Mixed-model ANOVA and multiple comparison tests (Interaction (Time*Treatment): (*p* < 0.0001); Treatments (DMSO/RLS0071/ACV): (*p* < 0.0001); and Time (days): (*p* < 0.0001)) and (**D**) one-way ANOVA (*p* < 0.0001) and independent *t*-tests (DMSO-RLS0071: *p* = 0.0465; DMSO-ACV: *p* < 0.0001; ACV-RLS0071: *p* < 0.0001); * *p* < 0.05, ** *p* < 0.01, *** *p* < 0.001, **** *p* < 0.0001. All error bars represent SEM.

Our results indicate that vehicle DMSO-treated animals exhibited a 0% survival rate within 14 days (Figure 1A). RLS-0071-treated animals showed a 53.3% rate of survivability p.i. compared to the vehicle-treated control animals across 14 days (*p* < 0.0001 indicated by Log-rank (Mantel–Cox) test). The DMSO-treated animals exhibited significantly increased infection severity and severely compromised health (Figure 1B,C). In addition, RLS-0071-treated animals demonstrated a significant reduction in the vesicle formation compared to control animals on days 9–14 p.i. (*p* < 0.01 and *p <* 0.001, multiple comparisons tests). We also observed healing of the lesions on the skin flank of animals treated with RLS-0071 post day nine (Figure 1B). As expected, animals treated with ACV did not exhibit severe infection and demonstrated 100% survivability p.i. (Figure 1B,C). Analyzing the distribution of infection scores averaged per day for each group indicated that treatment with RLS-0071 significantly reduces infection severity across 14 days (*p* < 0.0001) compared with the DMSO-treated mice for which the infection scores peaked around day nine (Figure 1D).

### 3.4. Sequencing of ACV^R^-HSV-1 Thymidine Kinase, UL23, Gene

Mutations leading to the acquisition of acyclovir-resistance have been found in HSV-1 thymidine kinase gene, (UL23), which accounts for 95% of clinical isolates, or in polymerase gene (UL30), which accounts for 5% of clinical isolates [8,9,23]. Single nucleotide insertions, deletions, or substitutions cause a frameshift mutation resulting in the synthesis of non-functional/truncated thymidine kinase (TK). ACV^R^-HSV-1 strain 17+ was previously generated and verified in our laboratory [18].

**Figure 2:**
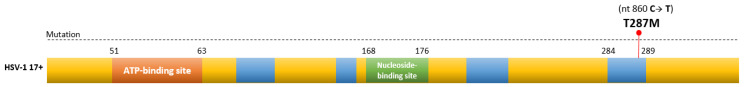
Schematic overview of conserved/active domains of viral thymidine kinase (TK). TK has 6 major conserved regions, an ATP-binding pocket (aa 51–63) composed of glycine-rich loop (red), a nucleoside-binding region (aa 168–177; green), and multiple highly conserved regions (blue), including aa 284–289. The viral TK gene of our ACV^R^-HSV-1 had a mutation at nt860 (cytosine to thymine), leading to a substitution mutation at aa 287 within the conserved region (threonine to methionine).

The TK gene in our ACV^R^-HSV-1 strain 17+ was sequenced to reveal possible mutations that cause drug resistance. The isolated viral TK sequence was compared to wild-type HSV-1 strain 17+ (NCBI Reference Sequence: NC_001806.2) using PubMed BLASTn and BLASTx (Figure S2). Our laboratory-generated ACV^R^-HSV-1 demonstrated a single-base nucleotide mutation in the viral UL23 gene from cytosine (C) to thymine (T) at nucleotide 860 within one of the highly conserved regions of TK [24,25]. This was reflected by a substitution mutation from threonine at amino acid (aa) 287 (T287) to methionine (T287M), resulting in a substitution mutation in the viral thymidine kinase gene (Figure 2).

### 3.5. RLS-0071 Protects BALB/cJ Mice against Cutaneous ACV^R^-HSV-1 Strain 17+ Infection

Due to the ongoing battle against drug-resistant HSV, we tested the effect of RLS-0071 against acyclovir-resistant infections. Female BALB/cJ mice (N = 8/treatment) were inoculated with 2.0 × 10^5^ PFU of ACV^R^-HSV-1 previously established in our laboratory [18]. The infected skin was treated with 10% DMSO, 14.4 mM RLS-0071, or 10 mM ACV formulated in 2.5% HEC gel. Each treatment was administered 1 h p.i. and continued twice daily (at 12-h intervals) for 14 days. The signs of disease at the inoculation site were scored per the appearance of vesicles.

**Figure 3:**
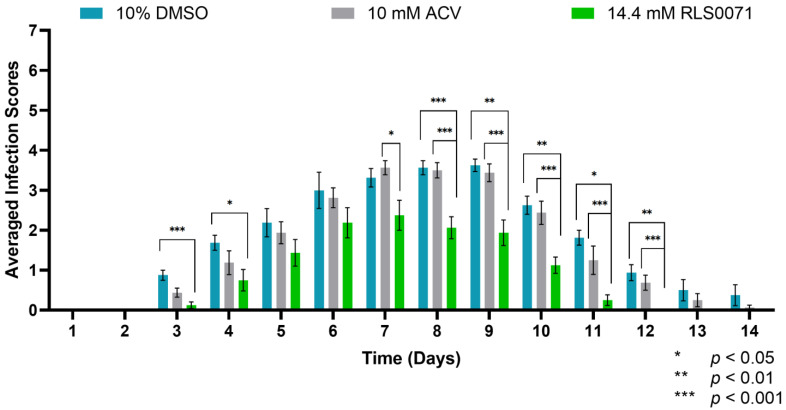
ACV^R^-HSV-1 infection scores of BALB/cJ mice following the application of 10% DMSO, 14.4 mM RLS-0071, or 10 mM ACV in 2.5% HEC gel (N = 8/group). Age-matched BALB/cJ mice were inoculated with 2.0 × 10^5^ PFU of ACV^R^-HSV-1 and treated. The mice were monitored for 14 days, and the averaged infection scores of animals in each treatment group were recorded. RLS-0071-treated BALB/cJ mice demonstrated a significant reduction in the appearance of vesicles compared to DMSO-treated animals. Mixed-model ANOVA and multiple comparison tests (Interactions (Time*Treatment): (*p* = 0.0017); Treatments (DMSO/RLS0071/ACV): (*p* < 0.0001); and Time (days): (*p* < 0.0001)); * *p* < 0.05; ** *p* < 0.01; *** *p* < 0.001. All error bars represent SEM.

Our results indicated 100% survivability of all animals regardless of treatment. This was expected because ACV-resistant mutants have been shown to have reduced pathogenicity in BALB/cJ mice, as judged by animal survival following infection [18,26]. Analyzing the interactions between BALB/cJ mice receiving DMSO or ACV revealed similar levels of infection, as no statistical significance was observed between the respective two treatment groups (*p* = 0.4655). On the other hand, RLS-0071-treated animals demonstrated a significant reduction in the formation of vesicles and erosions compared to DMSO-treated animals on days 3, 4, and 8-to-12 (*p <* 0.05, *p <* 0.01, and *p <* 0.001, indicated by multiple comparison tests). RLS-0071-treated mice also demonstrated a significant decrease in the vesicle formation compared to ACV-treated animals from days 7-to-12 (*p <* 0.05, *p <* 0.01, and *p <* 0.001, indicated by multiple comparison tests) (Figure 3). We observed that the infected vesicles on the skin of RLS-0071-treated mice healed completely by day 12. These results indicate that RLS-0071 exhibits efficacious effects on healing of the skin following ACV^R^ infection.

## 4. Discussion

In this study, we demonstrated that RLS-0071 significantly reduces the appearance of vesicles and erosions on the skin of GFP-HSV-1-infected BALB/cJ mice and significantly improves survivability when compared to HSV-1-infected animals receiving the control treatment. Infection scores in RLS-0071 animals were significantly lower compared to control-treated mice. The infection scores of BALB/cJ mice began decreasing around day nine, and healing of the infected sites was observed. Whereas infection scores of the control DMSO-treated mice continued to increase, by day 12, all control-treated animals succumbed. In contrast, HSV-1-infected animals receiving 10 mM of ACV did not demonstrate formation of infected vesicles, which is consistent with the effect of ACV seen in previous studies [18]. This effect of RLS-0071 is also consistent with our observations in animals infected with ACV-resistant HSV-1 strain 17+. RLS-0071-treated BALB/cJ mice demonstrated a significant reduction in the appearance of infected vesicles when compared to animals receiving control or ACV treatment across 14 days. The infection score of ACV^R^-infected animals treated with DMSO and ACV peaked around days seven to eight p.i., whereas the infection score for RLS-0071 did not peak, but rather, lesions healed completely by day 11 p.i.

The first phase of acute cutaneous infection caused by HSV-1 occurs in keratinocytes at the site of infection. As the virus enters the sensory neurons, it travels to the dorsal root ganglia (DRG), where it replicates. Following replication, the virus travels in an anterograde manner from the DRG back to the skin, inducing the second phase of viral growth, which results in zosteriform infection across the dermatome [6,27]. The role of neutrophils has been studied during viral infection, specifically their recruitment to skin flanks at the peak of infection [28,29]. Infected or damaged cells release pathogen-associated molecular patterns (PAMP), which trigger the release of inflammatory chemokines (Cxcl1/2/3) by resident macrophages and mast cells, leading to the recruitment of neutrophils to the infected sites [29,30]. The innate immune system is activated when pattern-recognition receptors detect viral PAMPs. The role of toll-like receptors (TLRs) in recognizing three classes of HSV PAMPs, such as viral proteins, DNA, and RNA, has been extensively reviewed [30,31,32]. At the cell surface, TLR2 senses viral glycoproteins B (gB) and gH/L, which activate nuclear factor κB pathway to induce expression of chemokines (C-X-C chemokine ligands) and pro-inflammatory cytokines (TNF-α, IL-6, IL-12). After entering the cell, endosomal TLR3/9 are activated by HSV nucleic acids, and PRRs (NOD-like receptors, melanoma differentiation-associated gene 5, interferon-inducible protein 16, and several helicases) recognize viral DNA and RNA in the cytoplasm. As a result, type I and III interferon (IFN) signaling is activated in human keratinocytes and infiltrating monocytes [31,32]. Previous studies have shown neutrophil accumulation below the infected epidermal layer; however, only a small number of neutrophils can migrate to the draining lymph nodes after T-cell priming five to seven days post infection, which is consistently seen within HSV-1-infected keratinocytes [33]. Hung et al. reported that although neutrophils in circulation undergo apoptosis within 24–36 h, HSV-1 can still be detected in neutrophils 36 h post exposure [34]. As the neutrophils are broken into apoptotic bodies, they are engulfed by macrophages. Live HSV-1 viruses within the engulfed apoptotic bodies can survive, evading the immune system and facilitating the spread of infection [34]. Thus, targeting the excessive infiltration of immune cells at the site of infection may reduce the site inflammation, thereby decreasing the appearance of surface vesicles and erosions. As we report in this paper, RLS-0071 has shown significant reduction in the appearance of vesicles on the skin of infected mice. We attributed the reduction of lesions to RLS-0071’s similar success in healing chronic diabetic wounds [16]. Cunnion et al. reported that RLS-0071 reduced inflammation, as observed by a reduction in activation of the complement system and leukocyte infiltration, after applied directly onto the skin of diabetic C57BL/Ks db/db male mice or when saturated in an acellular skin scaffold [16]. Hair et al. reported that within systemic lupus erythematosus (SLE) pathogenesis, RLS-0071 not only inhibited immune complex-initiated complement activation but also inhibited neutrophil extracellular trap (NET) formation in a dose-dependent fashion [14]. The inhibition of NET formation was suggested to occur following the inhibition of myeloperoxidase (MPO), which mediates NET formation by generating hypochlorous acid from hydrogen peroxide and chloride ions [14,15]. This would suggest that RLS-0071 may decrease excessive immune complex-initiated complement activation and accumulation of neutrophils by inhibiting NET formation on the skin of mice with wild-type and drug-resistant HSV-1 infection, thereby aiding in the healing process.

It was previously reported that a higher proportion of mutations causing resistance to acyclovir normally occur within the conserved regions of the ATP-binding site and or the nucleoside-binding site [25,35]. However, conserved amino acid regions spanning loci of aa 83–88, aa 216–222, aa 162–164, and aa 284–289 are not as affected [32]. Interestingly, sequencing our laboratory-generated, acyclovir-resistant HSV-1 strain 17+ revealed a single-base substitution mutation in the conserved region of thymidine kinase (TK) gene occurring at nucleotide 860, which resulted in a nucleotide change from cytosine of the wild-type HSV-1 17+ to thymine in the ACV^R^-HSV-1 strain 17+. Studies investigating ACV-resistance have often reported that 50% of HSV-1 drug resistance is attributable to a frameshift mutation in TK, whereas the majority of the mutations are triggered by amino acid substitutions following changes in a nucleotide that occurs in a non-conserved region (64.9%) as opposed to a conserved region (5.2%) [25]. We reported that the single-base nucleotide change in viral TK of ACV^R-^HSV-1 caused the substitution of threonine (T287) to methionine (T287M) in a conserved region of TK. A substitution mutation of T287M has also been previously reported by Sauerbrei et al. to occur in ACV-resistant clinical isolates of HSV-1 [24].

In conclusion, we report that RLS-0071 demonstrates the ability to decrease the appearance of vesicle formation on the flank of infected BALB/cJ mice. We propose that RLS-0071 may decrease excessive infiltration of leukocytes and complement activation at the site of infection, thereby promoting healing of lesions. Given these findings, RLS-0071 may have utility in conjunction with antiviral or virucidal compounds to aid in rapid wound healing of HSV-1 skin infections and potentially other pathology not limited to HSV-1 infection.

## Figures and Tables

**Table 1:** Thymidine kinase (TK) primers used with their respective sequences and melting temperatures.

Name	Melting Temperature	Sequences
Forward	54.3 °C	5′- CTT AAC AGC GTC AAC AGC G -3′
Reverse	54.5 °C	5′- CAC CCG TGC GTT TTA TTC TG -3′

**Table 2:** Zosteriform infection grading scale.

Grade	Skin Outcomes
0	no lesions
1, 2	local site lesions
3, 4, 5	distant site zosteriform lesions along the dermatome
6	progression to severely compromised health
7	mortality (succumbed to infection)

## Data Availability

The data presented in this study are available upon request from the corresponding author.

## Supplementary Materials

The following are available online at https://www.mdpi.com/article/10.3390/v13081422/s1, Table S1: Testing the antiviral effect of RLS-0071 using plaque assays, Table S2: Testing the virucidal effect of RLS-0071 using plaque assay, Figure S1: GFP-HSV-1 zosteriform infection scores of BALB/cJ mice (N = 5/treatment) following the application of 10% DMSO or 40% HIS buffer in 2.5% hydroxyethyl cellulose (HEC) gel, Figure S2: Analysis of ACV^R^-HSV-1 thymidine kinase gene sequence using PubMed BLASTn and BLASTx.
