# Supplementary material for: Peptide Inhibitor of Complement C1, RLS-0071, Reduces Zosteriform Spread of Herpes Simplex Virus Type 1 Skin Infection and Promotes Survival in Infected Mice
Source: Viruses. 2021 Jul 22;13(8):1422. doi: 10.3390/v13081422 (PMC8402672; doi:10.3390/v13081422)
Supplement: Supplementary file 1 [file viruses-13-01422-s001.zip › viruses-1270723-supplementary.pdf]

Supplementary Information

Peptide Inhibitor of Complement C1, RLS-0071, Reduces Zosteriform Spread of Herpes Simplex Virus Type 1 Skin Infection and Promotes Survival in Infected Mice

Maimoona S. Bhutta[1], Daniel G. Sausen[1], Kirstin M. Reed[1], Elisa S. Gallo[2], Pamela S. Hair[3], Brittany P. Lassiter[3], Neel K. Krishna[3], Kenji M. Cunnion[1,3,4,5,6] and Ronen Borenstein[1*]

[1] Department of Microbiology and Molecular Cell Biology, Eastern Virginia Medical School, Norfolk, VA 23507, USA.
[2] Board-certified dermatologist and independent researcher, Norfolk, VA 23507, USA.
[3] ReAlta Life Sciences, Inc., Norfolk, VA 23502, USA.
[4] Department of Pediatrics, Eastern Virginia Medical School, Norfolk, VA 23507, USA.
[5] Children's Specialty Group, 811 Redgate Avenue, Norfolk, VA 23507, USA.
[6] Children's Hospital of The King's Daughters, Norfolk, VA 23507, USA.

* Correspondence: Ronen Borenstein, **BorensR@evms.edu**.

Table S1. Testing the antiviral effect of RLS-0071 using plaque assays. 80-85% Vero cells were pretreated with varying concentrations of RLS-0071 or HIS Buffer (control treatment) for 1 hour at 37°C. After incubation, cells were infected with 0.1MOI of GFP-HSV-1 for 1 hour at 37°C in the presence of the respective treatments. Following infection, Vero cells were washed with DPBS 1x and incubated with fresh 199V media (without treatments) for 16 hours. The viruses from the infected cells were extracted and titered using plaque assay.

Treatment	0.1MOI GFP-HSV-1	Viral Titer (PFU/mL)
-	+	2.1×10^6
1 mM RLS-0071	+	5.2×10^6
2 mM RLS-0071	+	7.05×10^6
3 mM RLS-0071	+	7.85×10^6
4 mM RLS-0071	+	5.3×10^6
5 mM RLS-0071	+	4.8×10^6
1 mM HIS Buffer	+	3.9×10^6
2 mM HIS Buffer	+	2.9×10^6
3 mM HIS Buffer	+	7.5×10^6
4 mM HIS Buffer	+	4.95×10^6
5 mM HIS Buffer	+	3.35×10^6

Table S2. Testing the virucidal effect of RLS-0071 using plaque assay. 0.1MOI GFP-HSV-1 cell-free virus was incubated with varying concentrations of RLS-0071 or HIS Buffer (control treatment) for 1 hour at 37°C. Following incubation, viruses with the respective treatments were used to infect 80-85% confluent Vero cells for 1 hour at 37°C, then washed with DPBS 1x and incubated with fresh 199V media (without treatments) for 16 hours. The viruses from infected cells were extracted and titered using plaque assay.

Treatment	0.1MOI GFP-HSV-1	Viral Titer (PFU/mL)
-	+	2.5×10^6
1 mM RLS-0071	+	5.9×10^6
2 mM RLS-0071	+	7.05×10^6
3 mM RLS-0071	+	9.45×10^6
4 mM RLS-0071	+	2.85×10^6
5 mM RLS-0071	+	7.15×10^6
1 mM HIS Buffer	+	5.3×10^6
2 mM HIS Buffer	+	4.8×10^6
3 mM HIS Buffer	+	13.3×10^6
4 mM HIS Buffer	+	3.9×10^6
5 mM HIS Buffer	+	2.8×10^6

No difference observed in skin infection scores of BALB/cJ mice treated with DMSO or Histidine buffer

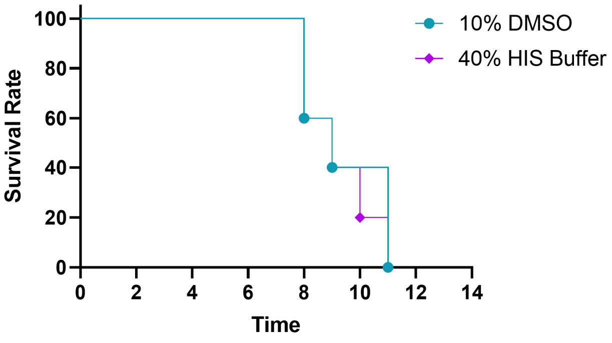

A

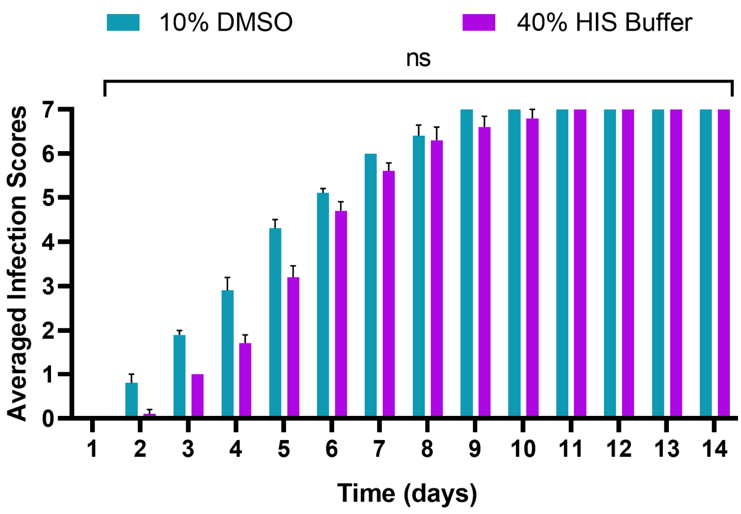

B

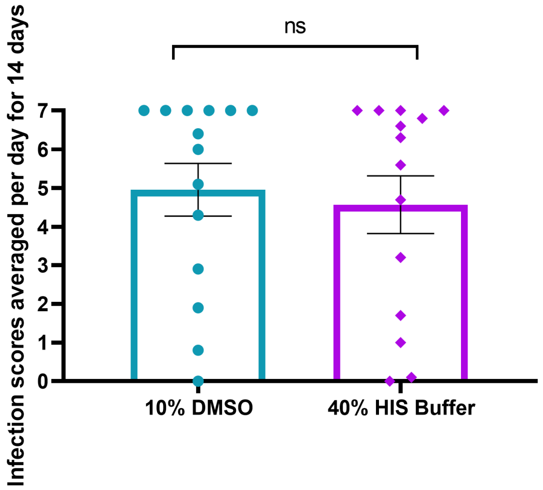

C

Figure S1. GFP-HSV-1 zosteriform infection scores of BALB/cJ mice (N = 5/treatment) following the application of 10% DMSO or 40% HIS buffer in 2.5% hydroxyethyl cellulose (HEC) gel. **(A)** Following inoculation with 6.0×10^4 PFU of GFP-HSV-1, BALB/cJ mice received respective treatments and were monitored for survival for 14 days. **(B)** Infection scores averaged in each treatment group across 14 days. **(C)** Distribution of averaged infection score of all animals per day for 14 days. **(B/C)** Student independent t-tests (2-tailed); *ns*- non-significant. All error bars represent SEM.

ORF of sequenced ACV^R-HSV-1 Thymidine kinase gene

ATGGCTTCGTACCCCTGCCATCAACACGCGTCTGCGTTCGACCAGGCTGCGCGTTCTCGCGGCCATAACAACCGACGTAC
GGCGTTGCGCCCTCGCCGGCAGCAAAAAGCCACGGAAGTCCGCCTGGAGCAGAAAATGCCCACGCTACTGCGGGTTTAT
ATAGACGGTCCCCACGGGATGGGGAAAACCACCACCACGCAACTGCTGGTGGCCCTGGGTTCGCGCGACGATATCGTCT
ACGTACCCGAGCCGATGACTTACTGGCGGGTGTTGGGGGCTTCCGAGACAATCGCGAACATCTACACCACACAACACCG
CCTCGACCAGGGTGAGATATCGGCCGGGGACGCGGCGGTGGTAATGACAAGCGCCCAGATAACAATGGGCATGCCTTA
TGCCGTGACCGACGCCGTTCTGGCTCCTCATATCGGGGGGGAGGCTGGGAGCTCACATGCCCCGCCCCCGGCCCTCACCC
TCATCTTCGACCGCCATCCCATCGCCGCCCTCCTGTGCTACCCGGCCGCGCGATACCTTATGGGCAGCATGACCCCCCAG
GCCGTGCTGGCGTTCGTGGCCCTCATCCCGCCGACCTTGCCCGGCACAAACATCGTGTTGGGGGCCCTTCCGGAGGACAG
ACACATCGACCGCCTGGCCAAACGCCAGCGCCCCGGCGAGCGGCTTGACCTGGCTATGCTGGCCGCGATTCGCCGCGTT
TATGGGCTGCTTGCCAATACGGTGCGGTATCTGCAGGGCGGCGGGTCGTGGCGGGAGGATTGGGGACAGCTTTCGGGGG
CGGCCGTGCCGCCCCAGGGTGCCGAGCCCCAGAGCAACGCGGGCCCACGACCCCATATCGGGGACATGTTATTTACCCT
GTTTCGGGCCCCCGAGTTGCTGGCCCCCAACGGCGACCTGTATAACGTGTTTGCCTGGGCTTTGGACGTCTTGGCCAAAC
GCCTCCGTCCCATGCACGTCTTTATCCTGGATTACGACCAATCGCCCGCCGGCTGCCGGGACGCCCTGCTGCAACTTACCT
CCGGGATGGTCCAGACCCACGTCACCACCCCAGGCTCCATACCGACGATCTGCGACCTGGCGCGCACGTTTGCCCGGGA
GATGGGGGAGGCTAACTGA

A

Alignment statistics				
**Score**	**Expect**	**Identities**	**Gaps**	**Strand**
2084 bits (1128)	0.0	1130 / 1131 (99%)	0 / 1131 (0%)	Plus / Minus

```
Query  1      ATGGCTTCGTACCCCTGCCATCAACACGCGTCTGCGTTCGACCAGGCTGCGCGTTCTCGC  60
              ||||||||||||||||||||||||||||||||||||||||||||||||||||||||||||
Sbjct  47803  ATGGCTTCGTACCCCTGCCATCAACACGCGTCTGCGTTCGACCAGGCTGCGCGTTCTCGC  47744

Query  61     GGCCATAACAACCGACGTACGGCGTTGCGCCCTCGCCGGCAGCAAAAAGCCACGGAAGTC  120
              ||||||||||||||||||||||||||||||||||||||||||||||||||||||||||||
Sbjct  47743  GGCCATAACAACCGACGTACGGCGTTGCGCCCTCGCCGGCAGCAAAAAGCCACGGAAGTC  47684

Query  121    CGCCTGGAGCAGAAAATGCCCACGCTACTGCGGGTTTATATAGACGGTCCCCACGGGATG  180
              ||||||||||||||||||||||||||||||||||||||||||||||||||||||||||||
Sbjct  47683  CGCCTGGAGCAGAAAATGCCCACGCTACTGCGGGTTTATATAGACGGTCCCCACGGGATG  47624

Query  181    GGGAAAACCACCACCACGCAACTGCTGGTGGCCCTGGGTTCGCGCGACGATATCGTCTAC  240
              ||||||||||||||||||||||||||||||||||||||||||||||||||||||||||||
Sbjct  47623  GGGAAAACCACCACCACGCAACTGCTGGTGGCCCTGGGTTCGCGCGACGATATCGTCTAC  47564

Query  241    GTACCCGAGCCGATGACTTACTGGCGGGTGTTGGGGGCTTCCGAGACAATCGCGAACATC  300
              ||||||||||||||||||||||||||||||||||||||||||||||||||||||||||||
Sbjct  47563  GTACCCGAGCCGATGACTTACTGGCGGGTGTTGGGGGCTTCCGAGACAATCGCGAACATC  47504

Query  301    TACACCACACAACACCGCCTCGACCAGGGTGAGATATCGGCCGGGGACGCGGCGGTGGTA  360
              ||||||||||||||||||||||||||||||||||||||||||||||||||||||||||||
Sbjct  47503  TACACCACACAACACCGCCTCGACCAGGGTGAGATATCGGCCGGGGACGCGGCGGTGGTA  47444

Query  361    ATGACAAGCGCCCAGATAACAATGGGCATGCCTTATGCCGTGACCGACGCCGTTCTGGCT  420
              ||||||||||||||||||||||||||||||||||||||||||||||||||||||||||||
Sbjct  47443  ATGACAAGCGCCCAGATAACAATGGGCATGCCTTATGCCGTGACCGACGCCGTTCTGGCT  47384

Query  421    CCTCATATCgggggggAGGCTGGGAGCTCACATGCCCCGCCCCCGGCCCTCACCCTCATC  480
              ||||||||||||||||||||||||||||||||||||||||||||||||||||||||||||
Sbjct  47383  CCTCATATCGGGGGGGAGGCTGGGAGCTCACATGCCCCGCCCCCGGCCCTCACCCTCATC  47324

Query  481    TTCGACCGCCATCCCATCGCCGCCCTCCTGTGCTACCCGGCCGCGCGATACCTTATGGGC  540
              ||||||||||||||||||||||||||||||||||||||||||||||||||||||||||||
```

```
Sbjct  47323  TTCGACCGCCATCCCATCGCCGCCCTCCTGTGCTACCCGGCCGCGCGATACCTTATGGGC  47264

Query  541    AGCATGACCCCCCAGGCCGTGCTGGCGTTCGTGGCCCTCATCCCGCCGACCTTGCCCGGC  600
              ||||||||||||||||||||||||||||||||||||||||||||||||||||||||||||
Sbjct  47263  AGCATGACCCCCCAGGCCGTGCTGGCGTTCGTGGCCCTCATCCCGCCGACCTTGCCCGGC  47204

Query  601    ACAAACATCGTGTTGGGGGCCCTTCCGGAGGACAGACACATCGACCGCCTGGCCAAACGC  660
              ||||||||||||||||||||||||||||||||||||||||||||||||||||||||||||
Sbjct  47203  ACAAACATCGTGTTGGGGGCCCTTCCGGAGGACAGACACATCGACCGCCTGGCCAAACGC  47144

Query  661    CAGCGCCCCGGCGAGCGGCTTGACCTGGCTATGCTGGCCGCGATTCGCCGCGTTTATGGG  720
              ||||||||||||||||||||||||||||||||||||||||||||||||||||||||||||
Sbjct  47143  CAGCGCCCCGGCGAGCGGCTTGACCTGGCTATGCTGGCCGCGATTCGCCGCGTTTATGGG  47084

Query  721    CTGCTTGCCAATACGGTGCGGTATCTGCAGGGCGGCGGGTCGTGGCGGGAGGATTGGGGA  780
              ||||||||||||||||||||||||||||||||||||||||||||||||||||||||||||
Sbjct  47083  CTGCTTGCCAATACGGTGCGGTATCTGCAGGGCGGCGGGTCGTGGCGGGAGGATTGGGGA  47024

Query  781    CAGCTTTCGGGGGCGGCCGTGCCGCCCCAGGGTGCCGAGCCCCAGAGCAACGCGGGCCCA  840
              ||||||||||||||||||||||||||||||||||||||||||||||||||||||||||||
Sbjct  47023  CAGCTTTCGGGGGCGGCCGTGCCGCCCCAGGGTGCCGAGCCCCAGAGCAACGCGGGCCCA  46964

Query  841    CGACCCCATATCGGGGACATGTTATTTACCCTGTTTCGGGCCCCCGAGTTGCTGGCCCCC  900
              |||||||||||||||||| |||||||||||||||||||||||||||||||||||||||||
Sbjct  46963  CGACCCCATATCGGGGACACGTTATTTACCCTGTTTCGGGCCCCCGAGTTGCTGGCCCCC  46904

Query  901    AACGGCGACCTGTATAACGTGTTTGCCTGGGCTTTGGACGTCTTGGCCAAACGCCTCCGT  960
              ||||||||||||||||||||||||||||||||||||||||||||||||||||||||||||
Sbjct  46903  AACGGCGACCTGTATAACGTGTTTGCCTGGGCTTTGGACGTCTTGGCCAAACGCCTCCGT  46844

Query  961    CCCATGCACGTCTTTATCCTGGATTACGACCAATCGCCCGCCGGCTGCCGGGACGCCCTG  1020
              ||||||||||||||||||||||||||||||||||||||||||||||||||||||||||||
Sbjct  46843  CCCATGCACGTCTTTATCCTGGATTACGACCAATCGCCCGCCGGCTGCCGGGACGCCCTG  46784

Query  1021   CTGCAACTTACCTCCGGGATGGTCCAGACCCACGTCACCACCCCAGGCTCCATACCGACG  1080
              ||||||||||||||||||||||||||||||||||||||||||||||||||||||||||||
Sbjct  46783  CTGCAACTTACCTCCGGGATGGTCCAGACCCACGTCACCACCCCAGGCTCCATACCGACG  46724

Query  1081   ATCTGCGACCTGGCGCGCACGTTTGCCCGGGAGATGGGGGAGGCTAACTGA  1131
              |||||||||||||||||||||||||||||||||||||||||||||||||||
Sbjct  46723  ATCTGCGACCTGGCGCGCACGTTTGCCCGGGAGATGGGGGAGGCTAACTGA  46673
```

B

Score	Expect	Method	Identities	Positives	Gaps	Frame
767 bits (1980)	0.0	Compositional matrix adjust.	375 / 376 (99%)	375 / 376 (99%)	0 / 376 (0%)	+1

```
Query  1      MASYPCHQHASAFDQAARSRGHNNRRTALRPRRQQKATEVRLEQKMPTLLRVYIDGPHGM  180
              MASYPCHQHASAFDQAARSRGHNNRRTALRPRRQQKATEVRLEQKMPTLLRVYIDGPHGM
Sbjct  1      MASYPCHQHASAFDQAARSRGHNNRRTALRPRRQQKATEVRLEQKMPTLLRVYIDGPHGM  60

Query  181    GKTTTTQLLVALGSRDDIVYVPEPMTYWRVLGASETIANIYTTQHRLDQGEISAGDAAVV  360
              GKTTTTQLLVALGSRDDIVYVPEPMTYWRVLGASETIANIYTTQHRLDQGEISAGDAAVV
Sbjct  61     GKTTTTQLLVALGSRDDIVYVPEPMTYWRVLGASETIANIYTTQHRLDQGEISAGDAAVV  120

Query  361    MTSAQITMGMPYAVTDAVLAPHIGGEAGSSHAPPPALTLIFDRHPIAALLCYPAARYLMG  540
              MTSAQITMGMPYAVTDAVLAPHIGGEAGSSHAPPPALTLIFDRHPIAALLCYPAARYLMG
Sbjct  121    MTSAQITMGMPYAVTDAVLAPHIGGEAGSSHAPPPALTLIFDRHPIAALLCYPAARYLMG  180
```

```
Query  541    SMTPQAVLAFVALIPPTLPGTNIVLGALPEDRHIDRLAKRQRPGERLDLAMLAAIRRVYG  720
              SMTPQAVLAFVALIPPTLPGTNIVLGALPEDRHIDRLAKRQRPGERLDLAMLAAIRRVYG
Sbjct  181    SMTPQAVLAFVALIPPTLPGTNIVLGALPEDRHIDRLAKRQRPGERLDLAMLAAIRRVYG  240

Query  721    LLANTVRYLQGGGSWREDWGQLSGAAVPPQGAEPQSNAGPRPHIGDMLFTLFRAPELLAP  900
              LLANTVRYLQGGGSWREDWGQLSGAAVPPQGAEPQSNAGPRPHIGD LFTLFRAPELLAP
Sbjct  241    LLANTVRYLQGGGSWREDWGQLSGAAVPPQGAEPQSNAGPRPHIGDTLFTLFRAPELLAP  300

Query  901    NGDLYNVFAWALDVLAKRLRPMHVFILDYDQSPAGCRDALLQLTSGMVQTHVTTPGSIPT  1080
              NGDLYNVFAWALDVLAKRLRPMHVFILDYDQSPAGCRDALLQLTSGMVQTHVTTPGSIPT
Sbjct  301    NGDLYNVFAWALDVLAKRLRPMHVFILDYDQSPAGCRDALLQLTSGMVQTHVTTPGSIPT  360

Query  1081   ICDLARTFAREMGEAN  1128
              ICDLARTFAREMGEAN
Sbjct  361    ICDLARTFAREMGEAN  376
```

C

Figure S2. Analysis of ACV^R-HSV-1 thymidine kinase gene sequence using PubMed BLASTn and BLASTx. **(A)** Open reading frame of sequenced acyclovir-resistant HSV-1 thymidine kinase gene. **(B)** Nucleotide sequence alignment generated using ORF of viral TK gene and NCBI Human alphaherpesvirus 1 strain 17, complete genome (Sequence ID: BK012101.1); match with the range of 46673 to 47803. The mutation in ACV^R-HSV-1 TK gene (query 1) occurring at nt 860 (C→ T) is highlighted in yellow. **(C)** Amino acid sequence analysis using BLASTx of mutated TK of ACV^R-HSV-1 with wild-type thymidine kinase [Human alphaherpesvirus 1] (NCBI Sequence ID: YP_009137097.1), with length of 376aa and 1 possible match. The mutation sequence (in query 1) occurring at aa 287 (T→ M) is highlighted in yellow.
